# White Matter Microstructure Associated with the Antidepressant Effects of Deep Brain Stimulation in Treatment-Resistant Depression: A Review of Diffusion Tensor Imaging Studies

**DOI:** 10.3390/ijms232315379

**Published:** 2022-12-06

**Authors:** Giulia Cattarinussi, Hossein Sanjari Moghaddam, Mohammad Hadi Aarabi, Letizia Squarcina, Fabio Sambataro, Paolo Brambilla, Giuseppe Delvecchio

**Affiliations:** 1 Department of Neuroscience (DNS), University of Padova, 35128 Padua, Italy; 2 Padova Neuroscience Center, University of Padova, 35128 Padua, Italy; 3 Department of Psychiatry, Roozbeh Psychiatric Hospital, Tehran University of Medical Sciences, Tehran 13185/1741, Iran; 4 Department of Pathophysiology and Transplantation, University of Milan, 20122 Milan, Italy; 5 Department of Neurosciences and Mental Health, Fondazione IRCCS Ca’ Granda Ospedale Maggiore Policlinico, 20122 Milan, Italy

**Keywords:** treatment-resistant depression, deep brain stimulation, white matter, structural connectivity, diffusion tensor imaging

## Abstract

Treatment-resistant depression (TRD) is a severe disorder characterized by high relapse rates and decreased quality of life. An effective strategy in the management of TRD is deep brain stimulation (DBS), a technique consisting of the implantation of electrodes that receive a stimulation via a pacemaker-like stimulator into specific brain areas, detected through neuroimaging investigations, which include the subgenual cingulate cortex (sgCC), basal ganglia, and forebrain bundles. In this context, to improve our understanding of the mechanism underlying the antidepressant effects of DBS in TRD, we collected the results of diffusion tensor imaging (DTI) studies exploring how WM microstructure is associated with the therapeutic effects of DBS in TRD. A search on PubMed, Web of Science, and Scopus identified 11 investigations assessing WM microstructure in responders and non-responders to DBS. Altered WM microstructure, particularly in the sgCC, medial forebrain bundle, cingulum bundle, forceps minor, and uncinate fasciculus, was associated with the antidepressant effect of DBS in TRD. Overall, the results show that DBS targeting selective brain regions, including the sgCC, forebrain bundle, cingulum bundle, rectus gyrus, anterior limb of the internal capsule, forceps minor, and uncinate fasciculus, seem to be effective for the treatment of TRD.

## 1. Introduction

Major depressive disorder (MDD) is a disabling psychiatric disorder characterized by low mood, anhedonia, guilt, and neurovegetative symptoms, such as fatigue, loss of appetite or weight, and insomnia (American Psychiatric Association, DSM-5-TR). The 1-year prevalence of MDD is approximately 6%, although it varies considerably across countries [1], whereas its lifetime prevalence ranges from 10 to 20%; moreover, MDD is twice as common in women as in men [2,3]. Although for most patients MDD is episodic, the course of the illness is highly variable, and the duration, the number, and the pattern of episodes changes considerably between subjects [4]. Nonetheless, antidepressants represent the first line of treatment for MDD, with psychotherapy used as add-on treatment [5]. Notably, approximately 30% of patients with MDD fail to achieve remission with two or more adequate dose–duration antidepressants from different classes, and are referred to as having “treatment-resistant depression” (TRD) [6], which is characterized by lower acute remission rates and increased probability of relapse [7]. In addition, compared with treatment-responsive MDD, patients with TRD present more frequently a history of childhood adversities, negative life events, comorbid personality disorders, higher medical comorbidities, and increased risk of suicide [8,9]. Therefore, the identification of effective therapies is paramount for this disabling disorder. In this regard, several therapeutic strategies have been proposed for TRD, spanning from pharmacological treatments, including antipsychotics [10], mood stabilizers [11], and esketamine [12], to neurostimulation treatments, such as vagus nerve stimulation (VNS), electroconvulsive therapy (ECT), transcranial magnetic stimulation (TMS), and deep brain stimulation (DBS) [13]. Among these, in the last few years, DBS has evolved to become a promising and effective strategy in the management of TRD, considering its relatively favorable side effect profile and the vast array of potential stimulation parameters [14]. In DBS surgery, the electrodes are stereotactically implanted into specific brain areas, where stimulation is provided via a pacemaker-like stimulator that delivers an electrical stimulation [15]. Neuroimaging studies have provided evidence that MDD is characterized by structural and functional alterations in numerous brain areas, such as the cingulate cortex, dorsolateral and dorsomedial prefrontal cortex, striatum, thalamus, and insula [16,17,18]. These investigations have guided the choice of targets for DBS, which include the subgenual cingulate cortex (sgCC), ventral capsule/ventral striatum, nucleus accumbens, lateral habenula, inferior thalamic peduncle, medial forebrain bundles, and the bed nucleus of the stria terminalis [19]. The exact mechanism of DBS is still unknown, although it has been hypothesized that the suppression of gamma oscillations and facilitation of theta-gamma coupling by DBS is mediated by both sgCC activation of inhibitory circuits and an increase in plasticity in the frontal cortex [20], ultimately leading to the normalization of abnormal network connectivity [21]. Furthermore, in recent years, the results from diffusion tensor imaging (DTI) provided a useful tool not only in the definition of surgical targets for DBS [22,23], but also in the study of WM tracts in relation to treatment response [24] This is because DTI provides useful information on the orientation and integrity of white matter (WM) tracts in TRD [25], and allows us to investigate the changes in WM and connectivity between frontal and subcortical structures following DBS [26,27]. It is a relatively sensitive neuroimaging technique that assesses water molecule diffusion in extracellular space within the tissue, and has the potential to provide useful information regarding alterations in the microstructure of the WM [28]. Fractional anisotropy (FA), mean diffusivity (MD), axial diffusivity (AD), and radial diffusivity (RD) are four indices that are derived based on the DTI approach for the purpose of quantifying the tensors that are included within each voxel, and can be indicative of microstructural markers [29,30]. FA is indicative of the degree of anisotropic movement of water. MD quantifies the average movement of water in three-dimensional directions. Moreover, RD and AD measure the movement of water molecules perpendicular to and alongside the nerve fibers, respectively. Lower microstructural integrity is associated with decreased FA and increased MD, RD, or AD. In TRD, reduced FA in the right anterior limb of the internal capsule, the body of the corpus callosum, bilateral external capsule, in the left limbic lobe uncus, left middle frontal gyrus and right cerebellum posterior lobe, bilateral inferior fronto-occipital fasciculus, bilateral inferior longitudinal fasciculus, bilateral superior longitudinal fasciculus, forceps major and forceps minor, and bilateral cingulum have been described in patients with TRD compared with healthy controls (HC) [31]. In this context, with this review, we aim at providing a comprehensive overview of WM microstructure associated with the therapeutic effects of DBS in TRD, with the final goal of clarifying the potential applications and advantages of the use of this technique in patients with TRD.

## 2. Results

### 2.1. Demographic, Clinical, and Stimulation Characteristics of Included Studies

**Figure 1:**
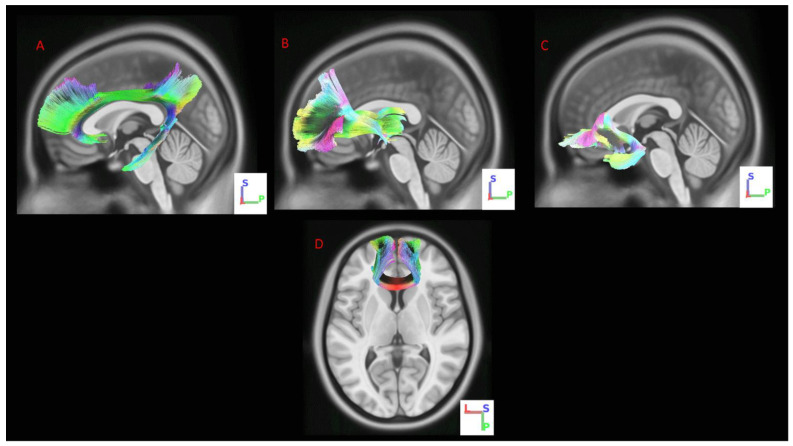
White matter tracts associated with the antidepressant effects of TRD: (**A**) cingulum [26,35,36,37]; (**B**) anterior thalamic radiation [24]; (**C**) uncinate fasciculus [26,35,37]; (**D**) forceps minor [35,36,37]. L, left; P, posterior; S, superior.

A total of 11 studies conducted between 2012 and 2020 investigating the association between presurgical WM measurements and response to DBS treatment were included in this review. The sample size ranged from 2 [32] to 24 [33] patients. Seven studies [22,24,26,27,34,35,36] included only patients with MDD, while one study [37] assessed both patients with MDD and bipolar disorder (BD). Three studies [32,33,38] did not specify the type of underlying disorder. Patients were middle-aged men and women in most studies. When reported, the average age of onset and duration of current major depression episode ranged from 15.2 to 25.8 years old and 5.7 to 64.1 months. Treatment response was assessed using the Hamilton Depression Rating Scale (HDRS) [39], Montgomery–Åsberg Depression Rating Scale (MADRS) [40], Beck’s Depression Inventory (BDI) [41], or the Global Assessment of Functioning (GAF) [42]. The majority of included studies applied DBS to the sgCC [32,34,35,36,37] or medial forebrain bundle [22,27,33]. The remaining investigations applied DBS to the ventral capsule/ventral striatum [38], posterior rectus gyrus [26], and ventral anterior limb of the internal capsule [24]. The methods and key findings of the studies are detailed in Table 1. The most commonly reported WM tracts are depicted in Figure 1.

### 2.2. Investigating the Role of WM Alterations in DBS Therapeutic Effect Using Tractography

Except for one study [24], the others used 3T MRI machines with a b-value of 1000. They all applied tractography (Table 1). Overall, the investigations used four major approaches to conduct tractography: (a) seed-to-target probabilistic tractography between pre-defined target regions and patient-specific volume of activated tissue (VAT) [32,34,36], defined as the spatial extent of the electrical field delivered by the lead to the anatomical structures near the selected target [43,44]; (b) whole-brain probabilistic tractography with VAT as the seed [22,27,35,37,38]; (c) bundle-specific tractography, considering some specific WM tracts for the analysis [24,33,35]; (d) whole-brain unconstrained probabilistic tractography [26].

### 2.3. WM Tracts Mediating the Effects of Subgenual Cingulate Cortex DBS on TRD

Of the 11 included studies, 5 applied DBS to the sgCC [32,34,35,36,37]. Overall, the results of these studies demonstrated that three primary fibers might mediate the effects of sgCC DBS on TRD, including cingulum bundle, forceps minor, and uncinate fasciculus. In particular, a pivotal study by Riva-Posse et al. (2014) reported that, compared to non-responders, responders to DBS showed robust connectivity in bilateral and medial aspect of the uncinate fasciculus connecting VAT to the medial frontal cortex, the cingulum bundle connecting the VAT to the rostral and dorsal anterior cingulate cortex and middle cingulate cortex, as well as short descending midline fibers connecting the VAT to subcortical nuclei, including the nucleus accumbens, caudate, putamen, and anterior thalamus. Moreover, the patients that converted from non-responders at six-month follow-up to responders at 2-year follow-up gained connectivity from VAT to the above-mentioned regions through these fibers [37]. In accordance, Tsolaki et al. (2017) used seed-to-target probabilistic tractography to investigate the connectivity of VAT to bilateral medial prefrontal cortex via forceps minor and uncinate fasciculus, ipsilateral ventral striatum, and anterior cingulate cortex in two subjects with TRD. They found that VAT showed structural connectivity to all four target regions in the responder patient, while there was no such connectivity in the non-responder [32]. Conversely, the findings of Clark et al. (2020) did not agree with these two studies. Specifically, these authors reported that the whole-brain probabilistic tractography, with VAT as the seed, showed more limited connectivity in non-responders compared to responders from VAT to the medial frontal and to the temporal lobe. In addition, projections to the striatum were limited in both groups, but were more prominent in the non-responders. The authors created a common tract map for responders and non-responders, in which all voxels were shared by all group members. The cingulum bundle was not identified in the common tracts map of either responders or non-responder groups at either time point. However, the bundle-specific analysis showed different results at 6-month and 1-year follow-ups. In particular, responders had significantly more VAT overlap with the cingulum bundle than non-responders at 6 months, but significantly less VAT overlap with the forceps minor tract at 1 year. The profile of other tracts, including uncinate fasciculus and frontostriatal projections, did not differ between responders and non-responders [35]. In contrast to previous studies, Howell et al. (2019) investigated the WM pathways of interest surrounding the sgCC, including the forceps minor, cingulum bundle, uncinate fasciculus, and frontal pole, with a different approach. In a sample of six patients, they assessed whether axonal activation could predict time to response. Their findings showed that cingulum bundle and forceps minor were activated in most patients. Using regression analysis, they demonstrated that four regressors (forceps minor, left cingulum bundle, right cingulum bundle, and right frontal pole (FP)) explained 99% of the variation in the patient’s time to stable response. However, activation of the right cingulum bundle alone explained 84% of the variance in time to stable response, and the addition of forceps minor, left cingulum bundle, and right FP did not contribute significant information to the linear model. Therefore, they concluded that increased predictive power from the inclusion of forceps minor, left cingulum bundle, and right FP in the linear model was likely because of overfitting [36]. Lastly, the study by Riva-Posse et al. (2019) assessed the role of WM connections in heart rate alterations. A significant relationship was found between the estimated structural connectivity of the left sgCC VAT to the middle cingulate cortex and the change in heart rate. Notably, it was found that the greater the structural connectivity between the sgCC VAT and the middle cingulate cortex (MCC), the more the heart rate increased for specific stimulation trials [34].

### 2.4. WM Tracts Mediating the Effects of Medial Forebrain Bundle DBS on TRD

Three studies applied DBS to the medial forebrain bundle [22,27,33]. Two studies by Fenoy et al. were conducted on an overlapping group of patients [22,27]. In the first study, contrary to non-responder patients, the three responder patients presented strong connectivity between the target location of the active electrode contact points and the medial prefrontal cortex [22]. Similar results were observed at a one-year follow-up [27]. In contrast, Coenen et al. (2019) found no significant relationships between microstructural measures of superolateral medial forebrain bundle and treatment response. Nonetheless, a positive relationship was reported between treatment response and enlargement of WM in left frontopolar superolateral medial forebrain bundle [33].

### 2.5. WM Tracts Mediating the Effects of DBS on Other Regions in Patients with TRD

The study by Lujan et al. (2012) assessed the axonal activation after DBS on the ventral anterior internal capsule and ventral striatum. They analyzed axonal activation by determining the extracellular voltages along each axon model (228,960 axon models for the 14 patient-specific DBS electric fields) by interpolating the patient-specific 3D electric fields onto each axon model compartment. Their results demonstrated that five pathways passing through the ventral anterior internal capsule and coursing lateral and medial to the ventral striatum, or dorsal and lateral to the nucleus accumbens, were common among 75% of responder patients, while one pathway adjacent to the ventromedial surface of the dorsal striatum was common among 75% of non-responders [38]. Conversely, Acolla et al. (2016) applied whole-brain unconstrained probabilistic tractography after DBS on the posterior rectus gyrus. They demonstrated that the probability of projections reaching the medial prefrontal cortex through the forceps minor and nucleus accumbens, or the anterior caudate through the uncinate fasciculus, was higher in the responder patient than in the average non-responders. Conversely, lower connectivity probability was found for tracts reaching the anterior cingulate cortex and middle cingulate cortex through the cingulum bundle in the responder patient [26]. Lastly, Liebrand et al. (2020) employed DBS on the ventral anterior limb of the internal capsule and reconstructed the superolateral medial forebrain bundle and anterior thalamic radiations for all patients using probabilistic tractography. There was a significant relationship between the average distance between tracts of interest and VAT and percentage change in HDRS [24].

## 3. Discussion

In this study, we reviewed all the available evidence to investigate whether patterns of WM microstructural integrity are associated with treatment response after DBS in TRD patients. In general, despite some inconsistencies in the results, it seems that DBS applied to different anatomical targets located in cortical, cingulate, and subcortical areas might stimulate different nodes of an interconnected network. This hypothesis is based on overall findings showing that treatment response after DBS with different targets, i.e., the cingulum bundle, forceps minor, uncinate fasciculus, medial forebrain bundle, and sgCC, is associated with the microstructural connectivity of similar WM tracts. However, future studies with graph theoretical analysis could better elucidate the network connectivity after DBS for TRD.

More in detail, the main targets of DBS in the included studies were the sgCC and the superolateral medial forebrain bundle. This is not surprising, as these regions play a key role in the pathophysiology of depression. Specifically, the sgCC is a crucial node with widespread connections to cortical and subcortical regions. Evidence from studies on primates has demonstrated that it is in reciprocal connection with the prefrontal, orbitofrontal, and dorsal cingulate cortices [45,46,47], as well as subcortical areas, such as the amygdala, thalamus, ventral striatum, and brainstem [46,48]. In addition, human studies using PET and fMRI have demonstrated the involvement of the sgCC in emotional processing in depressed individuals [49,50]. Conversely, the medial forebrain bundle is an important tract of the mesolimbic reward system, interconnecting the ventral tegmental area, hypothalamus, nucleus accumbens, and limbic lobe [51,52]. Notably, the ventral tegmental area is a critical hub within the dopaminergic reward system [53,54], and appears to modulate depressive symptoms [54]. Interestingly, it has been hypothesized that DBS effects on the medial forebrain bundle might lead to the recruitment of glutamatergic fibers from the medial prefrontal cortex to the ventral tegmental area, and it might therefore regulate ventral tegmental area dopaminergic activity [55]. In agreement, overall, the reviewed studies showed that stimulating various anatomical targets with DBS, including superolateral medial forebrain bundle, sgCC, and also the rectus gyrus, led to clinical improvement in patients with TRD. This suggests that aberrant electrical activity in these regions contributes to the development of TRD, and thus, acts as crucial hubs for the treatment of depression in patients with TRD.

In addition, from the reviewed studies emerged that other WM tracts seem to mediate the effects of DBS on TRD, including the cingulum bundle, forceps minor, and uncinate fasciculus, all structures that connect key neuroanatomical structures in cortical (prefrontal and temporal cortex), cingular, and subcortical (striatum) areas, and have been implicated in the pathophysiology of MDD. Specifically, the cingulum bundles are the main intrahemispheric association pathways connecting intrahemispheric structures with the middle cingulate cortex [56]. Notably, simultaneous intraoperative behavioral and tractography assessments demonstrated that the activation of the cingulum bundle leads to changes in interoception [57], which leads to the modulation of the rostral anterior cingulate cortex and anterior insular cortex [58], two key regions communicating through the subgenual cingulum bundle [59].

Furthermore, the subgenual cingulum bundle is also associated with the dorsal anterior cingulate cortex [60,61], which is an important target for the treatment of MDD [62]. In this regard, previous studies have shown that lesion severity of the cingulum bundle is a prognostic predictor of poor treatment response in depression [63], and that the microstructural integrity of the cingulum bundle is associated with better treatment outcomes after DBS in TRD [36]. Noteworthy, also, is that the forceps minor, which is the biggest WM tract connecting the medial forebrain to other brain regions, has been implicated in the pathophysiology of affective disorders [64], with previous evidence also showing that targeting this region has proven to be effective in reducing symptoms in TRD subjects [37]. Similarly, the uncinate fasciculus, which is the key fiber connecting the orbitofrontal to the temporal cortex [65], is also engaged in the processing and regulation of emotion [66,67]. Interestingly, DTI studies have described an impaired microstructural integrity of uncinate fasciculus in patients with MDD [68], indicating the role of this structure in the development of depression. However, in contrast to the cingulum bundle and forceps minor, the evidence regarding the role of the uncinate fasciculus in mediating the effects of DBS on depression is still limited.

Finally, the results of DBS–DTI studies suggest that treatment resistance was generally associated with less WM connectivity among crucial brain regions, while responder patients showed a more interconnected network. This evidence seems to indicate that a connected WM architecture might be needed for an optimal response after DBS for TRD.

Importantly, the results of the reviewed studies should be considered in light of several limitations, which may influence the generalizability of our conclusions. First, in 7 studies, the sample size was <10, and in the remaining 4, it ranged from 14 to 24. Sample size is a limitation in most DBS studies in psychiatric patients, therefore, care must be taken when interpretating the results. Second, there was a significant heterogeneity in terms of methodologies (e.g., target regions of DBS and DBS protocols) and populations (e.g., diagnostic criteria for TRD, mixed diagnosis, heterogeneous medications regimens), which in turn might decrease the generalizability of the findings. Indeed, specifically for medications, previous studies have shown that the use of antidepressants is associated with altered WM microstructural integrity [69]. Third, the retrospective nature of several of the included studies represents an intrinsic limitation. Fourth, since DTI is an indirect measure of the structural integrity of WM, to date, it is not entirely clear how perturbations in DTI parameters reflect in the axonal structure of WM fiber bundles. Therefore, DTI results should be interpreted with caution.

## 4. Materials and Methods

### 4.1. Article Selection

**Figure 2:**
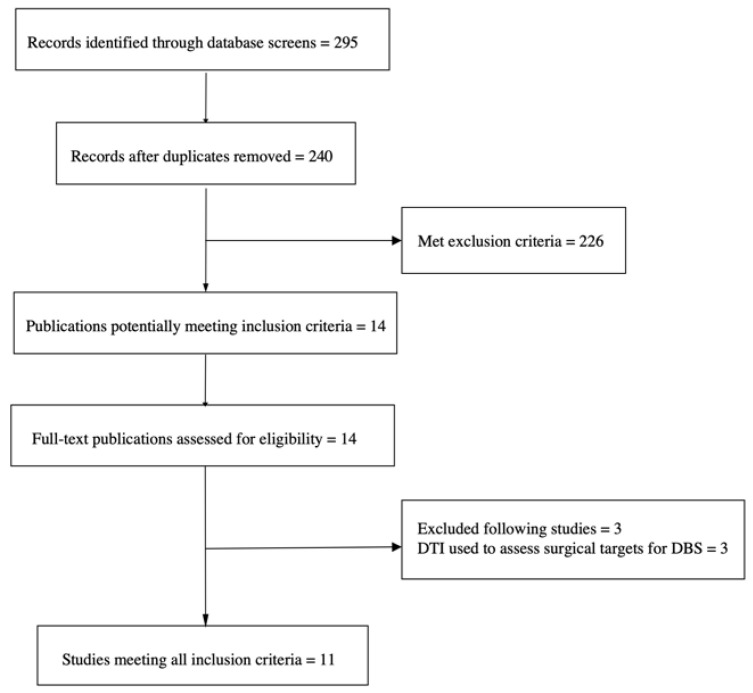
PRISMA flow chart of selection of publications for inclusion in review.

A systematic search on PubMed, Scopus, and Web of Science was conducted to identify all relevant studies published before January 2022 with no language restrictions. The keywords included the following terms: “treatment resistant depression” OR “resistant depression” OR “refractory depression” AND “Deep Brain Stimulation” OR DBS AND “Diffusion Tensor MRI” OR “Diffusion Tensor Magnetic Resonance Imaging” OR “DTI MRI” OR “Tractography” OR “white matter” OR “microstructural damage” OR “fractional anisotropy” OR “Diffusion Tensor Imaging”. We also traced the references of the relevant articles to find additional eligible studies. We included articles if they (1) explored WM using DTI or diffusion tensor MRI; (2) were conducted on individuals with TRD undergoing DBS. We excluded articles that (1) were conducted on subjects with disorders other than TRD (e.g., Parkinson’s disorder, obsessive compulsive disorder); (2) did not perform DBS. We also excluded letters to the editor, commentaries, and meeting abstracts. Data selection was conducted in accordance with the Preferred Reporting Items for Systematic Reviews and Meta-Analyses (PRISMA) guidelines [70]. Two authors (GC and HSM) independently performed the eligibility assessment. Our search resulted in 295 articles, 240 of which remained after removing duplicates. After title/abstract screening, 224 papers did not meet our inclusion criteria, and were excluded. After full-text reading, 3 articles were excluded, thus resulting in 11 eligible DTI studies (Figure 2). Details of the characteristics of the selected studies are shown in Table 2.

### 4.2. Data Extraction

One author (HSM) extracted the following data, which were checked by a second author (GC): (a) characteristics of the sample (age, gender, age of onset, duration of current depressive symptoms, medication profile, duration of follow-up); (b) diagnostic and psychopathological assessments; (c) technical specification of image acquisition; (d) DBS location; (e) key WM findings; (f) clinical outcomes.

## 5. Conclusions

In conclusion, the evidence that emerges from the reviewed studies demonstrates that DBS targeting the sgCC, superolateral medial forebrain bundle, rectus gyrus, and anterior limb of the internal capsule is associated with significant improvement in depressive symptoms. More importantly, the evidence suggests that the cingulum bundle, forceps minor, uncinate fasciculus, medial forebrain bundle, and sgCC seem to be the main WM structures mediating the beneficial effects of DBS on depressive symptoms in TRD. Nonetheless, future studies are indispensable to achieve enough clinical detail and statistical power to further our understanding on the association between WM structure and response to DBS.

## Figures and Tables

**Table 1:** Methodological approach and key findings of the DTI studies exploring the effects on WM of DBS in TRD.

Study	MRI (Tesla/b-Value)	Method of Analysis for DTI	DBS Location	DBS Stimulation Parameter	Key Findings
[38]	3/1000	Streamline tractography (a 60 × 60 × 60 mm ROI encompassing all sites of therapeutic stimulation) Electric field finite element Axonal activation model	VC/VS	4–7 V, 60–210 μs, 100–130 Hz	Active pathways common to 75% of responder patients: five pathways passed through the ventral anterior internal capsule and coursed lateral and medial to the VS or dorsal and lateral to the NA. Active pathways common to 75% of non-responder patients: one pathway was adjacent to the ventromedial surface of the dorsal striatum and followed a general trajectory.
[37]	3/1000	Activation volume probabilistic tractography (seed: VAT)	SCC	6–10 mA, 91 μs, 130 Hz	6-month and 2-year responders: three bilateral WM pathways were common to responders: (1) bilateral FM and medial aspect of the UF connecting the activation volume to the medial frontal cortex; (2) the CB connecting the activation volume to the rostral and dorsal ACC and MCC; (3) short descending midline fibers connecting the activation volume to subcortical nuclei including the NA, caudate, putamen, and anterior thalamus. 6-month and 2-year non-responders: lacked the connections mentioned above, with shared tracts failing to reach the frontal poles and body of the CB and with fewer connections to subcortical areas.
[26]	3/1000	Whole-brain unconstrained probabilistic tractography	Posterior gyrus rectus	90 μs, 130 Hz, 5 V	The probability of projections reaching medial PFC through the FM and NA, or anterior caudate through the UF, was higher in the responder patient than in the average non-responder. Conversely, lower connectivity probability was found for tracts reaching the ACC and MCC through the CB in the responder patient.
[22]	3/1000	Probabilistic tractography (seed: VAT)	Medial forebrain bundle	125 Hz, 75 μs, 2–3 mA	The three responder patients have strong connectivity between the target location of the active electrode contact points and the medial PFC. The non-responder patient has limited, sparse connectivity between the seed region and the PFC on planning images.
[32]	3/1000	Seed-to-target probabilistic tractography (seed: VAT, target: bilateral medial PFC via FM and UF, ipsilateral ventral striatum and ACC)	SCC	8 mA, 130 Hz, 91 μs	The structural connectivity of estimated VAT in the responder demonstrated connectivity with all four targets, whereas in the non-responder, there was <1% probability of connectivity (i.e., number of streamlines per seed voxel) with the ventral striatum.
[27]	3/1000	Probabilistic tractography (seed: VAT)	Medial forebrain bundle	125 Hz, 75 μs, 2–3 mA	At 1-year follow-up: all but the non-responder had strong connectivity between the target location of the active contact and the OFC. The non-responder patient had limited, sparse connectivity between the seed region and the PFC.
[33]	3/1000	slMFB-based volume analysis Whole-brain volume analysis Tractography of slMFB	slMFB	1–3 mA, 130 Hz, 60 μs	No significant relationships between microstructural measures of slMFB and treatment response were found. Positive relationship between treatment response and enlargement of SM in left frontopolar slMFB terminals. This enlargement was confined to WM and not to associated cortical regions.
[36]	3/1000	Seed-to-target probabilistic tractography (streamlines from tractography were used to approximate the trajectories and locations of axons within WM pathways of interest surrounding the SCC region; seeds: FM, CB, UF, and frontal pole)	SCC	4 V, 60–90 μs, 130 Hz	Activated axons within WM pathways of interest: DBS directly activated both CBs in every subject except one patient, where only the right CB was activated. FM was also activated in most patients. Compared to FM and CB, the two other pathways were activated to a much lesser degree. Correlations of axonal responses with clinical outcomes: four regressors (FM, left CB, right CB, and right FP) explained 99% of the variation in the patient’s time to stable response. Activation of the right CB, alone explained 84% of the variance in time to stable response, and the addition of FM, left CB, and right FP did not contribute significant information to the linear model.
[34]	3/1000	Seed-to-target probabilistic tractography between patient-specific VAT and pre-defined target (seed: MCC, medial frontal, amygdala, caudate, putamen, thalamus, NA, OFC, raphe nuclei, and VTA bilaterally)	SCC	6 mA, 130 Hz, 90 μs	Greater the structural connectivity between the SCC VAT and the MCC, the more the heart rate increased for specific stimulation trials. No significant relationship was found between the estimated structural connectivity of the SCC VAT to any other potential connected targets and the change in heart rate.
[35]	3/1000	Whole-brain probabilistic tractography (seed: VAT) Bundle-specific tractography analysis (tract of interest: CB, UF, FM, and frontostriatal projections)	SCC	90–450 μs, 130 Hz, 4–8 V	Whole-brain probabilistic tractography: The common tract map showed that all responders shared tracts projecting to the medial frontal pole and the temporal lobe. Non-responders showed a more limited tract profile, with projections more confined to the local area and lacking the lateral projections to the medial frontal and temporal lobes. Projections to the striatum were limited in both groups but were more prominent in the non-responders. The cingulum bundle was not identified in the common tract map of either responders or non-responders. Bundle-specific tractography analysis: At 6 months: responders had significantly more VAT overlap with the CB than non-responders. Responders and non-responders did not significantly differ in VAT overlap with the FM, the UF, or the frontostriatal projections. At 1 year: responders had significantly less VAT overlap with the FM tract compared to non-responders. Responders and non-responders did not significantly differ in VAT overlap with the CB, the UF, or the frontostriatal projections.
[24]	3/600	Probabilistic tractography (seed: VTA for slMFB and anterior thalamus for ATR)	Ventral anterior limb of the internal capsule	3.5–7.3 V, 60–120 μs, 130–190 Hz	Tracts of interest (slMFB and ATR) were reconstructed for all patients. There was a significant relationship between the average distance between tracts of interest and DBS contacts and percentage response. The VAT analysis showed that only 35 out of 56 (62.5%) bundles (2 bundles by 2 hemispheres by 14 patients) were located within the VAT. More specifically, the ATR was in VAT range in both hemispheres for 9 patients (11 left, 10 right), whereas the VAT covered the slMFB in both hemispheres in only 2 patients (5 left, 9 right). The average distance of both tracts to the VAT was significantly associated with the percentage change in HDRS.

ACC/MCC, anterior/middle cingulate cortex; ATR, anterior thalamic radiation; BA, Brodmann area; BDI, Beck’s Depression Inventory; CB, cingulate bundle; FP, frontal pole; FM, forceps minor; HDRS, Hamilton Depression Rating Scale; MADRS, Montgomery–Åsberg Depression Rating Scale; MCC, middle cingulate cortex; MRI, magnetic resonance imaging; NA, nucleus accumbens; OFC, orbitofrontal cortex; PFC, prefrontal cortex; ROI, region of interest; SCC, subcallosal cingulate cortex; slMFB, superolateral branch of medial forebrain bundle; UF, uncinate fasciculus; VAT, volume of activated tissue; VC/VS, ventral capsule/ventral striatum; VTA, ventrotegmental area; WM, white matter.

**Table 2:** Socio-demographic and clinical characteristics of the individuals included in DTI studies exploring the effects on WM of DBS in TRD.

Author Year	TRD Patients (M/F)	Diagnosis	Age (Years)	Age of Onset (Years)	Duration of Current MDE (Months)	Medications	Assessment Tool (Baseline Score)	Duration of Follow-Up	Clinical Outcomes
[38]	7 (2/5)	NR	42.42 (13.28)	NR	NR	NR	HDRS 32.0 (4.08) MADRS 30.42 (4.50) GAF 45.14 (2.60)	26.42 (9.79) months	HDRS, MADRS, and GAF improvements from baseline: 66.8% (43.7%), 78.5% (34.3%), and 34.8% (20.7%). Five remitters and two non-responders.
[37]	17 (7/10)	10 MDD, 7 BD	42.0 (8.9)	19.9 (7.8)	64.1 (53.7)	NR	HDRS 23.9 (0.7) BDI 38.4 (2.1) GAF 33.9 (1.7)	6 months and 2 years	6-month: 7 responders and 10 non-responders 2-year: 13 responders and 2 non-responders
[26]	5 (4/1)	5 MDD	45.2 (14.4)	25.0 (8.83)	NR	3 AP, 3 AD, 1 MS, 2 anxiolytics	HDRS 28.6 (3.13) BDI 41.0 (10.36)	3 and 6 months	One responder and four non-responders
[22]	4 (2/2)	4 MDD	46.3 (8.9)	16.5 (3.4)	6.3 (2.1)	Medicated	HDRS 39.8 (2.2) MADRS 34 (2.9)	1 week and 6 months	Three responders at week 1 and two responders at month 6
[32]	2	NR	NR	NR	NR	NR	MADRS	NR	One responder and one non-responder
[27]	6 (2/4)	6 MDD	50.2 (10.2)	15.2 (6.3)	5.7 (2.1)	Medicated	HDRS 39.5 (1.8) MADRS 35 (2.8)	1 week, 6 months, 1 year	Three responders at week 1 and four responders at 1 year
[33]	24	NR	NR	NR	NR	NR	MADRS	6 months and 1 year	NR
[36]	6 (2/4)	6 MDD	54.1 (8.9)	25.83 (7.16)	48.0 (44.25)	Medicated	HDRS 21.58 (1.80)	1 year	All were responders, with time to stable response being 98.66 (68.67) days
[34]	9 (2/7)	9 MDD	46.2 (8.29)	21.11 (11.61)	36.67 (20.0)	NR	HDRS 22.53 (2.78)	NR	NR
[35]	19 (10/9)	19 MDD	Responders: 42.2 (15.4) Non-responders: 50.2 (13.4)	NR	Responders: 19.3 (23.3) Non-responders: 33.0 (20.1)	NR	HDRS Responders: 23.2 (2.82) Non-responders: 24.1 (4.9)	6 months and 1 year	6 months: 9 responders and 10 non-responders 1 year: 9 responders and 10 non-responders
[24]	14	14 MDD	18–65	NR	NR	NR	HDRS	416 (154) days	The treatment response was, on average, 7.4 points (-33%) on the HDRS, with seven responders

AD, antidepressants; AP, antipsychotics; BD, bipolar disorder; BDI, Beck’s Depression Inventory; DBS, deep brain stimulation; GAF, Global Assessment of Functioning; HDRS, Hamilton Depression Rating Scale; MADRS, Montgomery–Åsberg Depression Rating Scale; MDD, major depressive disorder; MDE, major depression episode; MS, mood stabilizers; NR, not reported; SCC, subcallosal cingulate cortex; slMFB, superolateral branch of medial forebrain bundle; TRD, treatment-resistant depression; VC/VS, ventral capsule/ventral striatum. Data are presented as the mean (SD).

## Data Availability

Not applicable.
